# National nutrition surveillance programmes in 18 countries in South-East Asia and Western Pacific Regions: a systematic scoping review

**DOI:** 10.2471/BLT.23.289973

**Published:** 2023-10-04

**Authors:** Remco Peters, Bai Li, Boyd Swinburn, Steven Allender, Zouyan He, Sim Yee Lim, Mary Chea, Gangqiang Ding, Weiwen Zhou, Phonesavanh Keonakhone, Maikho Vongxay, Souphaxay Khamphanthong, Rusidah Selamat, Azucena Dayanghirang, Ellen Abella, Filipe Da Costa, Saipin Chotivichien, Narttaya Ungkanavin, Mai Tuyet Truong, Son Duy Nguyen, Bee Koon Poh

**Affiliations:** a Centre for Exercise, Nutrition and Health Sciences, School for Policy Studies, University of Bristol, 8 Priory Road, Bristol BS8 1TH, England.; b School of Population Health, University of Auckland, Auckland, New Zealand.; c Faculty of Health, Deakin University, Geelong, Australia.; d School of Public Health, Guangxi Medical University, Nanning, China.; e Faculty of Health Sciences, Universiti Kebangsaan Malaysia, Kuala Lumpur, Malaysia.; f National Maternal and Child Health Centre, Ministry of Health, Phnom Penh, Cambodia.; g National Institute for Nutrition and Health, Chinese Center for Disease Control and Prevention, Beijing, China.; h National Nutrition Centre, Ministry of Health, Vientiane, Lao People’s Democratic Republic.; i Nutrition Division, Ministry of Health Malaysia, Kuala Lumpur, Malaysia.; j National Nutrition Council, Taguig City, Philippines.; k Office of the Prime Minister, Díli, Timor-Leste.; l Bureau of Nutrition, Ministry of Public Health, Nonthaburi, Thailand.; m National Institute of Nutrition, Hanoi, Viet Nam.

## Abstract

**Objective:**

To identify and analyse ongoing nutrition-related surveillance programmes led and/or funded by national authorities in countries in South-East Asian and Western Pacific Regions.

**Methods:**

We systematically searched for publications in PubMed® and Scopus, manually searched the grey literature and consulted with national health and nutrition officials, with no restrictions on publication type or language. We included low- and middle-income countries in the World Health Organization South-East Asia Region, and the Association of Southeast Asian Nations and China. We analysed the included programmes by adapting the United States Centers for Disease Control and Prevention’s public health surveillance evaluation framework.

**Findings:**

We identified 82 surveillance programmes in 18 countries that repeatedly collect, analyse and disseminate data on nutrition and/or related indicators. Seventeen countries implemented a national periodic survey that exclusively collects nutrition-outcome indicators, often alongside internationally linked survey programmes. Coverage of different subpopulations and monitoring frequency vary substantially across countries. We found limited integration of food environment and wider food system indicators in these programmes, and no programmes specifically monitor nutrition-sensitive data across the food system. There is also limited nutrition-related surveillance of people living in urban deprived areas. Most surveillance programmes are digitized, use measures to ensure high data quality and report evidence of flexibility; however, many are inconsistently implemented and rely on external agencies’ financial support.

**Conclusion:**

Efforts to improve the time efficiency, scope and stability of national nutrition surveillance, and integration with other sectoral data, should be encouraged and supported to allow systemic monitoring and evaluation of malnutrition interventions in these countries.

## Résumé

**Objectif:**

Identifier et analyser les programmes de surveillance nutritionnelle en cours, menés et/ou financés par les autorités nationales de pays des régions de l’Asie du Sud-Est et du Pacifique occidental.

**Méthodes:**

Nous avons effectué une recherche systématique de publications sur PubMed® et Scopus, mené des recherches manuelles dans la littérature grise et consulté des responsables nationaux de la santé et de la nutrition, sans restriction quant au type de publication ou à la langue. Nous avons inclus les pays à revenu faible et intermédiaire de la région de l'Organisation mondiale de la santé pour l'Asie du Sud-Est et de l'Association des nations de l'Asie du Sud-Est, ainsi que la Chine. Nous avons analysé les programmes inclus en adaptant le cadre d'évaluation de la surveillance de la santé publique des Centers for Disease Control and Prevention des États-Unis.

**Résultats:**

Nous avons identifié 82 programmes de surveillance dans 18 pays qui collectent, analysent et diffusent régulièrement des données sur la nutrition et/ou des indicateurs liés. Dix-sept pays ont mis en œuvre une enquête nationale périodique qui recueille exclusivement des indicateurs de résultats nutritionnels, souvent parallèlement à des programmes d'enquête liés à l'échelle internationale. La couverture des différentes sous-populations et la fréquence de surveillance varient considérablement d'un pays à l'autre. Nous avons constaté dans ces programmes une intégration limitée des indicateurs portant sur l'environnement alimentaire et le système alimentaire au sens large, et aucun programme ne surveille spécifiquement les données prenant en compte la nutrition dans l'ensemble du système alimentaire. La surveillance nutritionnelle des personnes vivant dans des zones urbaines défavorisées est également limitée. La plupart des programmes de surveillance existent sous une forme numérisée, recourent à des mesures pour garantir la qualité des données et font preuve de flexibilité, mais beaucoup sont mis en œuvre de manière incohérente et dépendent du soutien financier d'agences extérieures.

**Conclusion:**

Les efforts visant à améliorer l'efficacité temporelle, la portée et la stabilité de la surveillance nutritionnelle à l'échelle nationale, ainsi que l'intégration avec d'autres données sectorielles, devraient être encouragés et soutenus afin de permettre un suivi et une évaluation systémiques des interventions en matière de malnutrition dans ces pays.

## Resumen

**Objetivo:**

Identificar y analizar los programas de vigilancia en curso relacionados con la nutrición que dirigen o financian las autoridades nacionales de los países de las regiones de Asia Sudoriental y el Pacífico Occidental.

**Métodos:**

Se realizaron búsquedas sistemáticas de publicaciones en PubMed® y Scopus, búsquedas manuales en la literatura gris y consultas con funcionarios nacionales de salud y nutrición, sin restricciones de tipo de publicación ni de idioma. Se incluyeron países de ingresos bajos y medios de la Región de Asia Sudoriental de la Organización Mundial de la Salud y de la Asociación de Naciones de Asia Sudoriental y China. Para analizar los programas incluidos se adaptó el marco de evaluación de la vigilancia de la salud pública de los Centros para el Control y la Prevención de Enfermedades de Estados Unidos.

**Resultados:**

Se identificaron 82 programas de vigilancia en 18 países que recopilan, analizan y difunden repetidamente datos sobre nutrición o indicadores relacionados. Diecisiete países aplicaron una encuesta periódica nacional que recopila exclusivamente indicadores de resultados nutricionales, por lo general junto con programas de encuestas internacionales. La cobertura de las diferentes subpoblaciones y la frecuencia del seguimiento varían sustancialmente de un país a otro. Se halló una integración limitada de los indicadores del entorno alimentario y del sistema alimentario en general en estos programas y ningún programa supervisa específicamente los datos que tienen en cuenta la nutrición en todo el sistema alimentario. También es limitada la vigilancia relacionada con la nutrición de las personas que viven en áreas urbanas desfavorecidas. La mayoría de los programas de vigilancia están digitalizados, utilizan medidas para garantizar la alta calidad de los datos y presentan evidencias de flexibilidad; sin embargo, muchos se aplican de forma incoherente y dependen del apoyo financiero de organismos externos.

**Conclusión:**

Se deben fomentar y apoyar los esfuerzos para mejorar la eficiencia temporal, el alcance y la estabilidad de la vigilancia nacional de la nutrición, así como la integración con otros datos sectoriales, para permitir un seguimiento y una evaluación sistémicos de las intervenciones contra la malnutrición en estos países.

## ملخص

تحديد وتحليل البرامج الجارية لمراقبة التغذية والتي تتولاها و/أو تمولها السلطات الوطنية في دول منطقة جنوب شرق آسيا ومنطقة غرب المحيط الهادئ.

الطريقة أجرينا عمليات بحث منهجية عن المنشورات الموجودة في ®PubMed وScopus، وبحثنا يدويًا في المؤلفات غير الرسمية وتشاورنا مع مسؤولي الصحة والتغذية على المستوى الوطني وذلك دون وضع أي قيود على نوع المنشورات أو لغتها. أدرجنا الدول ذات الدخل المنخفض والدخل المتوسط الأعضاء في المكتب الإقليمي لمنظمة الصحة العالمية لجنوب شرق آسيا ورابطة أمم جنوب شرق آسيا والصين. كما قمنا بتحليل البرامج المدرجة عن طريق إجراء تعديلات على إطار عمل تقييم مراقبة الصحة العامة الذي وضعته مراكز مكافحة الأمراض والوقاية منها في الولايات المتحدة.

النتائج حددنا 82 برنامجًا للمراقبة في 18 دولةً تجمع بصفة متكررة البيانات المتعلقة بالتغذية و/أو المؤشرات المرتبطة بها وتحليلها ونشرها. أجرت 17 دولةً استبيانًا دوريًا على المستوى الوطني بفرض جمع مؤشرات تقتصر على نتائج التغذية وغالبًا ما تصاحبها البرامج المتعلقة بالاستبيان على الصعيد الدولي. تتباين تغطية الفئات السكانية الثانوية المختلفة ومعدل تكرار المراقبة بشكل جذري بين تلك الدول. وقد لاحظنا محدودية التكامل بين البيئة الغذائية والمؤشرات الأعم للنظام الغذائي في هذه البرامج وعدم وجود برامج تراقب البيانات تراعي أمور التغذية عبر النظام الغذائي على وجه التحديد. كما توجد أيضًا مراقبة محدودة لتغذية الأشخاص الذين يعيشون في المناطق الحضرية المحرومة. يتم تنفيذ معظم برامج المراقبة بوسائل رقمية، وتستخدم هذه البرامج معايير لضمان الجودة العالية للبيانات والإبلاغ عن دليل المرونة، ومع ذلك، فإن العديد منها يتم تطبيقه بشكل غير متسق ويعتمد على تلقي الدعم المالي من جهة خارجية.

الاستنتاج يجب تشجيع ودعم الجهود المبذولة لتحسين الكفاءة الزمنية، ونطاق المراقبة الوطنية للتغذية واستمرارها، وتضافر تلك الجهود مع البيانات الواردة من قطاعات مختلفة وذلك من أجل إتاحة الفرصة لمراقبة وتقييم التدخلات الرامية لمواجهة سوء التغذية في هذه الدول بشكل منهجي.

## 摘要

**目的:**

确定和分析在东南亚和西太平洋地区各国由国家官方机构领导和/或资助的正在进行的与营养相关的监测项目。

**方法:**

我们系统地检索了 PubMed® 和 Scopus 中的发表论文，人工检索了灰色文献，并咨询了国家卫生和营养官员，不限制出版物类型和语言。我们纳入了世界卫生组织东南亚区域和东南亚国家联盟的低收入和中等收入国家以及中国。我们通过调整美国疾病控制和预防中心的公共卫生监测评估框架来分析纳入的项目。

**结果:**

我们确定了 18 个国家的 82 个监测项目，这些项目多次收集、分析和传播营养和/或相关指标的数据。17 个国家定期开展专门收集营养结果指标的全国调查，通常与国际联系的调查项目一起进行。不同亚群的覆盖范围和监测频率在不同国家差别很大。我们发现，这些项目对粮食环境和更广泛的粮食系统指标的整合有限，而且没有任何项目专门监测整个粮食系统的营养敏感数据。对生活在城市贫困地区的人的营养监测也很有限。大多数监测项目都实现了数字化，并采取措施确保高数据质量并报告灵活性证据，然而，许多监测项目不能贯彻实施，需要依赖外部机构的财政支持。

**结论:**

应鼓励和支持改善国家营养监测的时间效率、范围和稳定性以及与其他领域数据相整合的工作，以便对这些国家的营养不良干预措施进行系统监测和评估。

## Резюме

**Цель:**

Определить и проанализировать текущие программы надзора за питанием, осуществляемые и (или) финансируемые национальными органами власти в странах Юго-Восточной Азии и западной части Тихоокеанского региона.

**Методы:**

Проведен систематический поиск публикаций в базах данных PubMed® и Scopus, ручной поиск в серой литературе, а также проведены консультации с представителями национальных органов здравоохранения и питания без ограничений по типу публикации и языку. В исследование включены страны с низким и средним уровнем дохода, входящие в регион Юго-Восточной Азии Всемирной организации здравоохранения и Ассоциацию государств Юго-Восточной Азии, а также Китай. Проведен анализ включенных программ на основе адаптации системы оценки надзора в области общественного здравоохранения, разработанной центрами по контролю и профилактике заболеваний (CDC) США.

**Результаты:**

Выявлено 82 программы надзора в 18 странах, которые осуществляют постоянный сбор, анализ и распространение данных о питании и (или) связанных с ним показателях. В семнадцати странах проведены национальные периодические опросы, в ходе которых собирались исключительно показатели качества питания, часто наряду с программами международных опросов. Охват различных подгрупп населения и частота мониторинга существенно различаются между странами. В этих программах наблюдается ограниченная интеграция показателей состояния продовольственной среды и более широких показателей продовольственной системы, и ни одна из программ не осуществляет специального мониторинга данных, учитывающих особенности питания в рамках всей продовольственной системы. Также проводится ограниченный надзор за питанием людей, проживающих в неблагополучных городских районах. Большинство программ надзора оцифрованы, для них предусмотрены меры, обеспечивающие высокое качество данных, и есть основания предполагать их гибкий характер, однако многие из них реализуются непоследовательно и зависят от финансовой поддержки со стороны внешних агентств.

**Вывод:**

Необходимо поощрять и поддерживать усилия по повышению эффективности, масштабов и стабильности национального надзора за питанием, а также его интеграцию с другими отраслевыми данными, чтобы обеспечить системный мониторинг и оценку мероприятий по борьбе с нарушениями питания в этих странах.

## Introduction

In south-east Asia, low- and middle-income countries have a high burden from all forms of malnutrition, such as underweight, wasting, stunting and micronutrient deficiencies, obesity and diet-related noncommunicable diseases.^1^^–^^3^ Malnutrition is causing the most diseases and premature deaths in this region, and is associated with social and economic burdens.^3^ Despite substantial progress in reducing the prevalence of undernutrition, most countries are not meeting the global targets on maternal, infant and young child nutrition indicators for 2025. Moreover, no countries are on track to curb adult and childhood obesity.^4^

There has been a call for a transformative shift in how we conceptualize, develop and evaluate nutrition interventions. The goal is to synergistically address shared factors of multiple malnutrition forms, often termed as double- or triple-duty interventions, across various societal subsystems to ensure maximum and sustainable impact.^5^ The typically viewed benchmark in evaluation research is randomized controlled trials, however, they face ethical and practical challenges. Moreover, they may not effectively address the dynamic and adaptive nature of population-level interventions rooted in a systems approach. Using long-term, government-led and/or funded national surveillance programmes offers a more appropriate and sustainable method for evaluating population-level systemic interventions.^6^^,^^7^ Therefore, understanding the scope and characteristics of nutrition-related surveillance programmes is an important initial step to assess countries’ capability to monitor and evaluate systemic interventions. This understanding can also help guide the development and improvement and capacity building actions.

Previous research shows the scope of commonly used nutrition surveillance methods in low- and middle-income countries.^8^ Several valuable global data repositories that collate pre-collected data on nutrition and wider food systems are also available.^9^^–^^11^ However, a comprehensive and up-to-date overview of ongoing national nutrition surveillance programmes in south-east Asian countries and China is currently lacking.

As part of the Systemic Actions to Reduce Malnutrition In All Its Forms in South-east Asian Countries and China (SYSTAM CHINA-SEACS International Consortium) project, we conducted a systematic scoping review with the aim of identifying and analysing ongoing, nutrition-related national surveillance programmes for Member States in the World Health Organization (WHO) South-East Asia and Western Pacific Regions, and of the Association of Southeast Asian Nations (ASEAN).

## Methods

Our systematic scoping review is based on the six-stage published framework.^12^ We followed the Preferred Reporting Items for Systematic Reviews and Meta-Analysis for Protocols (PRISMA-P) checklist.^12^^–^^14^ This protocol has been registered with the Open Science Framework.^15^

### Search strategy

We searched for relevant studies, reports and documents on currently ongoing nutrition surveillance programmes. We searched two online databases, PubMed® and Scopus, for relevant publications using a combination of key search terms: monitor*, survey*, surveillance, weight, nutrition*, diet*, food*, eating and health*. We searched the databases from January 2014 to 29 January 2022 for most countries, and for some countries from inception to 29 January 2022, and updated this search to 26 June 2023. More details are available in the online repository.^16^

For our manual search, we conducted both forward and backward reference searches of the identified articles in the database search to locate additional relevant publications.^12^ To ensure that all relevant information is extracted, we searched for methodological documents of surveillance programmes on government and programme websites and from institutional websites of international organizations, such as WHO.

We complemented our desk-based literature search with consultation meetings and email communications with senior nutrition officials at health ministries in study countries from February until September 2022. These officials have been responsible for the design, deployment or implementation of national nutrition (health) surveillance programmes, and/or are familiar with existing or the development of nutrition surveillance programmes in their countries.^17^ The officials were purposively invited through our own networks with Asian governments.

We continuously refined and expanded our literature search and evaluation criteria in response to earlier search and consultation results, where desk-based research (academic database and manual search) and expert consultation informed each other iteratively.^12^

### Eligibility criteria

Here we define a nutrition surveillance, or monitoring, programme as repeated collection, analysis, interpretation and dissemination of primary data on the anthropometric and biochemical nutrition outcomes (exclusively or embedded) and behavioural or food system indicators that influence anthropometric or biochemical nutrition outcomes.^18^^,^^19^ Such programmes are an important part of a wider nutrition information system.^7^ To achieve a broader understanding of national government-led nutrition-related surveillance, we included programmes operated by non-health sectors within the food system. These programmes must either feature nutrition outcome indicators or collect data intended to enhance nutrition. In this review, primary data refer to data that are collected first-hand for a specific programme or purpose.^7^

We covered ongoing nutrition surveillance programmes in 18 low- and middle-income countries, 11 WHO South-East Asia Region Member States and seven WHO Western Pacific Region Member States. Ten of these 18 countries are ASEAN Member States (Table 1). We included ASEAN Member States due to their sustained health and nutrition partnerships, which allow for local and regional research objectives to be collectively developed and achieved for maximum impact. We excluded the ASEAN Member State Singapore because it is classified as a high-income country. To provide a more comprehensive picture of the nutrition surveillance in south-east Asia, we also included other Member States of the WHO South-East Asia and Western Pacific Regions. This inclusion allows for wider comparisons of similarities and differences across countries that share contextual characteristics, and further promotes international learning and sharing of experiences.

**Table 1:** Anthropometry and blood pressure surveillance per population group, frequency of monitoring and country, study countries

Physical measurement, by country	Interval between rounds, years								
	Birth^a^	Children < 5 years	Children 5–9 years	Adolescents 10–19 years	Women of reproductive age	Pregnant women	Lactating women	Adult men	Elderly people
**Weight and height**									
Bangladesh^20^^–^^26^	1–6^b^	1–3^b^	Once	Once, 1–4,^b,d^ once^c^	3–5^b^	NA	3–5^b^	4–5^b^	Once
Bhutan^27^^–^^30^	7	7	NA	3–7^d^, once ^c^	3–7	NA	NA	3–7	NA
Brunei Darussalam^31^^–^^35^	NA	NA	12	12, 5^c^	5–6^b^	NA	NA	5–6^b^	12
Cambodia^36^	NA	4–5	NA	Once^d^	4–5	NA	4–5	NA	NA
China^37^^–^^49^	NA	Annually	1–3^b^	1–3^b^	1–3^b^	1–3^b^	1–3^b^	1–3^b^	1–3^b^
Democratic People’s Republic of Korea^50^^,^^51^	8	3–13^b^	NA	NA	NA	NA	NA	NA	NA
India^52^^–^^64^	1–7^b^	1–6^b^	2^b^	3–5,^b^ 1–6^b,d^	1–6^b^	NA	1–6^b^	1–6^b^	Once
Indonesia^65^^–^^71^	1–4^b^	Annually	3–5	3–5, 8^c^	3–5	NA	3–5	3–5	3–5
Lao People’s Democratic Republic^47^^,^^72^^–^^74^	5	Biannually	NA	7^c^	5	NA	NA	5	NA
Malaysia^48^^,^^75^^–^^79^	6	4	4	5	4–5	NA	NA	4–5	1–5
Maldives^80^^–^^82^	5	7	NA	5^c^	5–7^b^	NA	NA	5–7^b^	NA
Myanmar^83^^–^^86^	Once	2^b^	Once	Once, 9^c^	3–5^b^	NA	Once	3–5^b^	NA
Nepal^87^^–^^91^	1–4^b^	1–4^b^	NA	Once, 1–4,^b,d^ once^c^	1–4^b^	NA	5–6^b^	1–4^b^	NA
Philippines^92^^–^^98^	1–3^b^	Annually	3	3, once^c^	3	3	3	3	3
Sri Lanka^99^^–^^106^	4–6^b^	4–6^b^	5^b^	8,^c^ once^f^	1–5^b^	NA	1–9^b^	1–5^b^	NA
Thailand^107^^–^^110^	3–10	2,^b^ once^ d^	5–6, once^c^	5–6, 6–7^c^	5–6, once^c^	NA	5–6	5–6, once^c^	5–6, once^c^
Timor-Leste^111^^–^^114^	7	4^b^	NA	1–4,^b,d^ once^c^	1–4^b^	3–4^b^	2–4^b^	2–4^b^	NA
Viet Nam^115^^–^^121^	3–6	3^b^	NA	11,^d^ 6^c^	5–6^b^	NA	11	5–6^b^	NA
**Circumferences (mid-upper arm and/or waist–hip ratio)**									
Bangladesh^20^^–^^26^	NA	3^e^	Once	Once	3–5^b^	NA	Once	3–5^b^	Once
Bhutan^27^^–^^30^	NA	NA	NA	3–7^d^	3–7	NA	NA	3–7	NA
Brunei Darussalam^31^^–^^35^	NA	NA	12	12	5–6^b^	NA	NA	5–6^b^	12
Cambodia^36^	NA	Once^e^	NA	Once^d,e^	Once^e^	Once^e^	Once^e^	NA	NA
China^37^^–^^49^	NA	1–3^b^	1–3^b^	1–3^b^	1–3^b^	NA	3^b^	1–3^b^	1–3^b^
Democratic People’s Republic of Korea^50^^,^^51^	NA	Once^e^	NA	Once^d,f^	Once^f^	NA	Once^f^	NA	NA
India^52^^–^^64^	NA	Once	Once	4,^b,d^ once	2^b^	NA	4^b^	2^b^	NA
Indonesia^65^^–^^71^	NA	3–5^e^	3–5	3–5^e^	3–5^e^	3–5^e^	3–5^e^	3–5	3–5
Lao People’s Democratic Republic^47^^,^^72^^–^^74^	NA	NA	NA	NA	5	NA	NA	5	NA
Malaysia^48^^,^^75^^–^^79^	NA	NA	NA	NA	4–5	NA	NA	4–5	1–5
Maldives^80^^–^^82^	NA	NA	NA	NA	7–11	NA	NA	7–11	NA
Myanmar^83^^–^^86^	NA	2–6^b,e^	Once	Once	4–6^b^	5, once^e^	NA	5, once^e^	NA
Nepal^87^^–^^91^	NA	Once^e^	NA	NA	2–6	NA	NA	2–6	NA
Philippines^92^^–^^98^	NA	NA	NA	NA	3	NA	NA	3	3
Sri Lanka^99^^–^^106^	NA	NA	NA	NA	4–7	NA	NA	4–7	NA
Thailand^107^^–^^110^	NA	NA	NA	5–6	NA	NA	NA	NA	NA
Timor-Leste^111^^–^^114^	NA	7	NA	NA	Once, once^e^	Once^f^	Once^e^	Once	NA
Viet Nam^115^^–^^121^	NA	NA	NA	NA	6	NA	NA	6	NA
**Skinfold**									
Bangladesh^20^^–^^26^	NA	NA	NA	NA	NA	NA	NA	NA	NA
Bhutan^27^^–^^30^	NA	NA	NA	NA	NA	NA	NA	NA	NA
Brunei Darussalam^31^^–^^35^	NA	NA	NA	NA	NA	NA	NA	NA	NA
Cambodia^36^	NA	NA	NA	NA	NA	NA	NA	NA	NA
China^37^^–^^49^	NA	NA	2–4	2–4	2–4	NA	NA	2–4	2–4
Democratic People’s Republic of Korea^50^^,^^51^	NA	NA	NA	NA	NA	NA	NA	NA	NA
India^52^^–^^64^	NA	NA	5^b^	5^b^	Once	Once	Once	Once	Once
Indonesia^65^^–^^71^	NA	NA	NA	NA	NA	NA	NA	NA	NA
Lao People’s Democratic Republic^47^^,^^72^^–^^74^	NA	NA	NA	NA	NA	NA	NA	NA	NA
Malaysia^48^^,^^75^^–^^79^	NA	NA	NA	NA	NA	NA	NA	NA	NA
Maldives^80^^–^^82^	NA	NA	NA	NA	NA	NA	NA	NA	NA
Myanmar^83^^–^^86^	NA	NA	NA	NA	NA	NA	NA	NA	NA
Nepal^87^^–^^91^	NA	NA	NA	NA	NA	NA	NA	NA	NA
Philippines^92^^–^^98^	NA	NA	NA	NA	NA	NA	NA	NA	NA
Sri Lanka^99^^–^^106^	NA	NA	NA	NA	NA	NA	NA	NA	NA
Thailand^107^^–^^110^	NA	NA	NA	NA	NA	NA	NA	NA	NA
Timor-Leste^111^^–^^114^	NA	NA	NA	NA	NA	NA	NA	NA	NA
Viet Nam^115^^–^^121^	NA	NA	NA	NA	NA	NA	NA	NA	NA
**Blood pressure**									
Bangladesh^20^^–^^26^	NA	NA	NA	NA	1–4^b^	1–4^b^	1–6^b^	1–4^b^	Once
Bhutan^27^^–^^30^	NA	NA	NA	NA	3–7	3–7	NA	3–7	NA
Brunei Darussalam^31^^–^^35^	NA	NA	NA	NA	5–6^b^	NA	NA	5–6^b^	12
Cambodia^36^	NA	NA	NA	NA	NA	NA	NA	NA	NA
China^37^^–^^49^	NA	3^b^	1–3^b^	1–3^b^	1–3^b^	1–3^b^	1–3^b^	1–3^b^	1–3^b^
Democratic People’s Republic of Korea^50^^,^^51^	NA	NA	NA	NA	NA	NA	NA	NA	NA
India^52^^–^^64^	NA	NA	Once	2,^b,d^ once	2^b^	NA	4	2^b^	NA
Indonesia^65^^–^^71^	NA	NA	NA	NA	3–5	3–5	3–5	3–5	3–5
Lao People’s Democratic Republic^47^^,^^72^^–^^74^	NA	NA	NA	NA	5	5	NA	5	NA
Malaysia^48^^,^^75^^–^^79^	NA	NA	NA	NA	4–5	4–5	NA	4–5	Once
Maldives^80^^–^^82^	NA	NA	NA	NA	7–11	7–11	NA	7–11	NA
Myanmar^83^^–^^86^	NA	NA	NA	NA	3–5	3–5	NA	3–5	NA
Nepal^87^^–^^91^	NA	NA	NA	1–3^b,d^	1–3^b^	1–3^b^	5	1–3^b^	NA
Philippines^92^^–^^98^	NA	NA	NA	3	3	3	3	3	3
Sri Lanka^99^^–^^106^	NA	NA	NA	NA	4–7	NA	NA	4–7	NA
Thailand^107^^–^^110^	NA	NA	NA	5–6	NA	NA	NA	NA	NA
Timor-Leste^111^^–^^114^	NA	NA	NA	NA	Once	Once	NA	Once	NA
Viet Nam^115^^–^^121^	NA	NA	NA	NA	6	6	NA	6	NA

NA: not applicable.

^a^ Only weight from written record and/or respondent’s reporting.

^b^ Monitoring by multiple programmes.

^c^ Self-reported weight and height among school-going adolescents in Global School-Based Student Health Survey.

^d^ Only covers adolescents 15–19 years of age.

^e^ Only mid-upper arm circumference measurement.

Our selection of programmes and relevant publications was defined by the eligibility criteria as specified under the inclusion and exclusion criteria presented in Box 1.

Box 1 **Inclusion and exclusion criteria of nutrition surveillance programmes and related publications, in study countries**

***Inclusion criteria***

- We included a programme when it is ongoing, led and funded by a country’s governmental body and repeatedly collects, analyses, interprets and disseminates primary data on anthropometric, biochemical, behavioural and/or food system indicators relating to nutrition. Examples include large-scale, repeated surveys, and data from repeatedly used sentinel sites and educational/childcare settings. Primary data refers to data that are collected for surveillance purposes.^7^
- Related publications were included when these covered methodological information on one, or multiple ongoing nationally representative nutrition (and health) surveillance data collection programme(s).
- We included nutrition (-related) surveillance programmes that are ongoing, or with minimally one recently completed data collection round, that are conducted on a continuous and/or periodic basis.
- We included programmes that had implemented at least one data collection round, and are predicted to see future rounds.
- Programmes and related publications that we included had to collect primary data on anthropometric and/or nutrition-related indicators, and be operating in at least one of the Member States of WHO South-East Asia Region, the Association of Southeast Asian Nations and/or China. The programme could cover any age and demographic group.
- We included all types of literature (or study designs) that provide information relevant to the design, methods, findings, and impact on or information for, at least one surveillance programme, such as government reports and conference papers.
- Publications could be written in any language.

***Exclusion criteria***

- Organizational or researcher-led collections and analyses of nutrition and related data which do not form part of a larger surveillance programme were not included.
- Publications that focus on secondary use of data from nutrition surveillance programmes when official documentation on methodological and operational information is available.
- Nutrition and health surveillance programmes that were discontinued before 2022.
- Programmes and related publications that do not collect primary data on anthropometric and/or nutrition indicators.
- Programmes and related publications that do not include relevant information or data on surveillance programmes in the following countries: China, any Member States of the WHO South-East Asia Region, and/or the Association of Southeast Asian Nations. Programmes implemented in high-income countries were excluded.

WHO: World Health Organization.

### Study selection

We imported the results from the academic database search into Endnote X9 (Clarivate, London, England). Two researchers independently screened titles and abstracts using the specified eligibility criteria in the open-access web-based systematic reviewing application Rayyan (Rayyan, Cambridge, United States of America). We resolved disagreements following abstract screening through discussions to build consensus. To ensure a low number of false negatives, we screened a preliminary test set of 50–100 records.^12^ Three reviewers independently screened the full text of eligible publications. A fourth reviewer addressed discrepancies between the three reviewers at the full-text screening stage.

### Data extraction

We extracted data for each surveillance programme via a purposively developed form on the basis of components and operation characteristics that are listed in the updated United States Centers for Disease Control and Prevention (CDC) guidelines for public health surveillance programmes. The 2001 CDC updated guidelines are commonly applied, and intended to be universally applicable for describing and evaluating a large variety of different public health surveillance programmes.^122^

The data extraction form included the authors, publication date, publication title, type of primary surveillance, main objectives (which we categorized based on previous work)^123^^,^^124^ and the country where the programme is being implemented. We further disaggregated the extracted information according to: (i) collected data and used method; (ii) target population and sampling characteristics; (iii) programme and data management; (iv) ethical procedures; and (v) information to assist impact assessment. The extraction form is available in the online repository.^16^

### Data synthesis

To provide a comprehensive description of surveillance at a national and programme level (Box 2), we analysed six adapted attributes derived from the 2001 CDC updated guidelines,^122^ which are informed by a set of standards for evaluation (detailed description available in online repository).^16^^,^^126^ We also enquired with national health and nutrition officials to obtain missing information and verify our description. We did not identify any conflicting information between the different identified documents. We triangulated findings from the programme analysis, and consultations with officials to present the current state of nutrition (-related) surveillance in south-east Asia and China.

Box 2 **Selected attributes of included nutrition surveillance programmes^a^ in study countries**

***Data quality***

We reported on programme-level validated measures and quality assurance methods that were used to ensure data quality. We also report data completeness as an indicator of data quality.

***Flexibility***

We based our analysis of flexibility on whether the programme reports any evidence of accommodated changes to the programme methods and operation between data collection rounds with the purpose of incorporating relevant indicators and adapting to population’s nutritional needs.^125^

***Representativeness***

We analysed national and programme-level representativeness through identification of geographical locations, subpopulation groups, and accurate reporting of nutrition-related events. The latter refers to the notion whether the collected data can be disaggregated by sociodemographic variables important to nutrition.^126^

***Timeliness and simplicity***

The timeliness and simplicity of surveillance programmes refers to both national and individual programme structure, and ease of operation. Based on available information, we analysed whether the identified programme digitized their data collection, processing and dissemination during its most recent round. We also describe whether there are any overlapping years of indicator selection, any reported barriers to timely and simple implementation, and the central body and partnerships being responsible for surveillance programmes at country level.

***Stability***

We analysed stability by considering any mentioned issues during the collection, management and provision of data, and the consistency of data collection (were there any gaps between data collection?), and type of funding.

^a^ Our selection was guided by the adapted US Centers of Disease Control and Prevention guidelines^122^ on public health surveillance system evaluation.

## Results

**Fig. 1:**
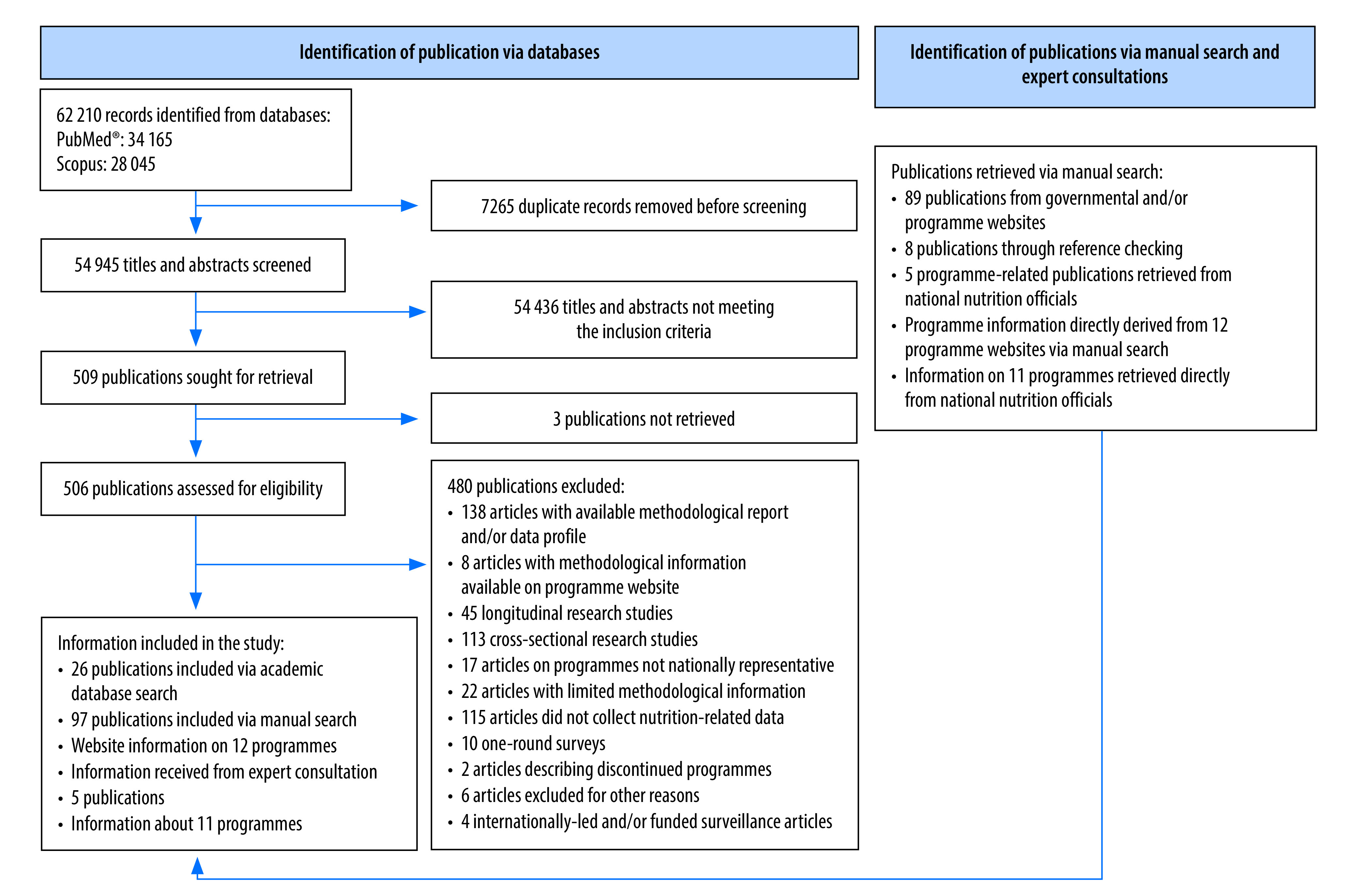
Flowchart of the selection of programme-relevant publications on nutritional surveillance programmes in study countries

We identified 54 945 unique papers through our academic database search; 26 of these met the inclusion criteria and we used them to derive programme information.^27^^,^^31^^,^^32^^,^^37^^–^^40^^,^^53^^–^^56^^,^^72^^,^^107^^,^^108^^,^^127^^–^^138^ For most programmes, we derived methodological information from 89 programme reports or factsheets,^20^^–^^26^^,^^28^^–^^30^^,^^33^^–^^36^^,^^43^^,^^50^^–^^52^^,^^57^^–^^62^^,^^65^^–^^69^^,^^73^^–^^78^^,^ ^80^^–^^95^^,^^99^^–^^106^^,^^109^^–^^119^^,^^139^^–^^157^ 12 programme and/or governmental websites^41^^–^^48^^,^^63^^,^^70^^,^^157^^–^^159^ and eight publications through our manual search of references.^49^^,^^64^^,^^71^^,^^96^^,^^120^^,^^123^^,^^160^^,^^161^ We also received information about 11 programmes and five related publications^79^^,^^97^^,^^98^^,^^121^^,^^162^ directly from national health and nutrition officials (Fig. 1).

Most identified programmes had a methodological report which was publicly available on the respective government or international agency website. Several reports were not available in English,^67^^,^^68^^,^^148^^,^^162^ in which case, the programme details were provided or verified by national nutrition and health officials, or complemented with peer-reviewed articles.

We provided an overview of the descriptive analysis of the included programmes and a summary of the main findings in Box 3. More detailed and programme-level information is available in the online repository.^16^

Box 3 **Summary of programmes and main findings of nutritional surveillance in study countries**

***Type of data collected***

- Seventeen countries have implemented national programmes that exclusively collect data on individual nutrition and diet-outcome indicators.
- No countries monitor indicators on all forms of malnutrition, or on food environment and wider food systems.

***State of nutrition surveillance***

- The majority of countries have digitized data collection, implemented comprehensive measures to promote data quality, and scaled-up or increased monitoring scope in comparison to its preceding round.
- Most programmes report higher than 80% response rates.
- All countries, if information is available, implement rigorous training and supervision practices prior to and during data collection.
- All countries have one or more programmes that adopted programmatic changes between data collection rounds.
- All countries have one or more programmes that included new indicators and subpopulation groups.
- Seven countries monitor anthropometry indicators among all age groups.
- Nine countries collect data on most micronutrient biomarkers.
- Five countries monitor individual dietary intake periodically among all age groups.
- While there are variations in terms of representativeness across countries, elderly people were commonly not represented in the monitoring of anthropometry and dietary data.
- Limited surveillance in urban deprived areas.
- All countries collect data which can be disaggregated by important nutrition-related population characteristics.
- Most programmes that collect nutrition and nutrition-related indicators were coordinated and implemented by countries’ health ministries.
- Most countries’ local surveillance programmes collected data on nutrition-outcome and/or diet-outcome indicators with consistent time intervals between rounds.
- Nine countries have an internally funded national surveillance programme that exclusively collects nutrition-outcome data.

### Identified programmes

We identified 82 nationally representative government-led and -funded surveillance programmes that repeatedly collect primary data on nutrition and/or diet outcome indicators. Some of the programmes implemented a first round within the last decade and may conduct, or have planned, future rounds. The programmes were either local or internationally linked. On a national level, health ministries (or an embedded research institute) coordinated and implemented most programmes that collect nutrition and nutrition-related indicators. The ministries use the collected data to inform the development and evaluation of national policies and nutrition programmes. To a lesser extent, programmes are designed to collect data that governments can leverage for informed decision-making and to monitor national objectives.

Of the 18 countries, eight exclusively collect data on individual nutrition and diet-outcome indicators through an established national nutrition surveillance programme, on a continuous basis, (Bangladesh, China, Indonesia, Lao People's Democratic Republic, Malaysia, Philippines, Thailand and Viet Nam).^21^^,^^38^^,^^48^^,^^49^^,^^65^^,^^75^^–^^77^^,^^92^^,^^119^^,^^121^^,^^149^^,^^150^^,^^162^ In nine countries, large-scale periodic national nutrition surveys were implemented (Bhutan, Brunei Darussalam, China, Democratic People's Republic of Korea, India, Sri Lanka, Timor-Leste, Thailand and Viet Nam).^28^^,^^33^^,^^50^^,^^54^^,^^58^^,^^99^^,^^111^^,^^116^^,^^120^^,^^135^^,^^156^ National micronutrient status surveys were used in three countries (Bangladesh, Nepal and Viet Nam);^26^^,^^87^^,^^157^^,^^163^ and in Myanmar, Sri Lanka and Viet Nam, national nutrition and micronutrient surveys were implemented.^83^^,^^103^^,^^163^ Further details on the key characteristics and indicators are available in the online repository.^16^

The type of collected data, used methods and monitoring frequency differed substantially across programmes and countries. We found that all countries periodically collect weight and height data, and most countries take waist and hip circumferences (Table 1). China, Indonesia, Lao People's Democratic Republic, Philippines and Viet Nam continuously collect weight and height data among children younger than 5 years.^38^^,^^65^^,^^92^^,^^119^^,^^121^ We only identified one programme in China and three in India that measure skinfold thickness.^40^^,^^57^^,^^58^

All countries periodically measure anaemia with varying monitoring frequency (ranging from a single round to 12-year intervals). Nine countries (Bangladesh, China, India, Indonesia, Myanmar, Nepal, Philippines, Sri Lanka and Thailand) also collect data on most other micronutrient deficiencies (Table 2; available at https://www.who.int/publications/journals/bulletin/). The Thai government implemented a surveillance programme that annually collects information on median urinary iodine among pregnant women, in households and antenatal care clinics.^162^ Most countries periodically collect biochemical and anthropometric data on diet-related noncommunicable diseases, mainly with the WHO STEPwise approach to noncommunicable disease risk factor surveillance.^164^

**Table 2:** Data collected on micronutrient deficiencies and biomarkers per population group, frequency of monitoring and country, study countries

Measurement, by country	Interval between rounds, years							
	Children < 5 years	Children 5–9 years	Adolescents 10–19 years	Women of reproductive age	Pregnant women	Lactating women	Adult men	Elderly people
**Micronutrient deficiencies and biomarkers**								
**Anaemia (haemoglobin level)**								
Bangladesh^22^^,^^24^^,^^26^^,^^157^	8	Once	Once	8	NA	NA	NA	NA
Bhutan^27^^–^^29^	7	NA	7	7	7	NA	NA	NA
Brunei Darussalam^31^^–^^34^	NA	NA	NA	12	NA	12	12	12
Cambodia^36^	4–7	NA	4–7^a^	4–7	4–7	4–7	NA	NA
China^37^^,^^38^^,^^40^^,^^49^	4–7^b^	3^b^	3^b^	3^b^	1^b^	3^b^	3^b^	3^b^
Democratic People's Republic of Korea^50^	Once	NA	Once^a^	Once	Once	Once	NA	NA
India^52^^,^^53^^,^^55^^–^^64^	2^a^	Once	Once	5	5	NA	5	NA
Indonesia^67^^,^^68^^,^^71^	3–5	3–5	3–5	3–5	3–5	3–5	3–5	3–5
Lao People's Democratic Republic ^47^^,^^72^^,^^73^	5	NA	5^a^	5	5	5	NA	NA
Malaysia^48^^,^^78^^,^^79^	NA	NA	4–5^a^	4–5	4–5	4–5	4–5	4–5
Maldives^80^^,^^81^	Once	NA	Once	Once	Once	Once	NA	NA
Myanmar^83^^–^^85^	1^b^	Once	1^a,b^	1^b^	Once	Once	NA	NA
Nepal^87^^–^^90^	5^b^	NA	5^a,b^	5^b^	5^b^	5^b^	NA	NA
Philippines^92^^,^^98^	3	3	3	3	3	3	3	3
Sri Lanka^99^^–^^103^^,^^105^^,^^154^	Once	Once	1,^b^ once^c^	NA	Once	NA	NA	NA
Thailand^107^^,^^108^^,^^162^	NA	NA	5–6	5–6	5–6	5–6	5–6	5–6
Timor-Leste^112^^,^^114^	NA	NA	3–4^b^	3–4^b^	7	3–4^b^	Once	NA
Viet Nam^116^^,^^117^^,^^120^^,^^163^	Once^d^	NA	Once^a,c^	Once^d^	Once^d^	Once^d^	NA	NA
**Vitamin A (modified relative dose response of serum retinol or retinol-binding protein) **								
Bangladesh^22^^,^^24^^,^^26^^,^^157^	8	One round	One round	8	NA	NA	NA	NA
Bhutan^27^^–^^29^	NA	NA	NA	NA	NA	NA	NA	NA
Brunei Darussalam^31^^–^^34^	NA	NA	NA	NA	NA	NA	NA	NA
Cambodia^36^	Once^e^	NA	Once^a,e^	Once^e^	Once^e^	Once^e^	NA	NA
China^37^^,^^38^^,^^40^^,^^49^	3	3	3	3	3	3	3	3
Democratic People's Republic of Korea^50^	NA	NA	NA	NA	NA	NA	NA	NA
India^52^^,^^53^^,^^55^^–^^64^	Once	Once	Once	NA	NA	NA	NA	NA
Indonesia^67^^,^^68^^,^^71^	NA	NA	NA	NA	NA	NA	NA	NA
Lao People's Democratic Republic^47^^,^^72^^,^^73^	NA	NA	NA	NA	NA	NA	NA	NA
Malaysia^48^^,^^78^^,^^79^	NA	NA	NA	NA	NA	NA	NA	NA
Maldives^80^^,^^81^	NA	NA	NA	NA	NA	NA	NA	NA
Myanmar^83^^–^^85^	Once	Once	Once^a^	Once	NA	NA	NA	NA
Nepal^87^^–^^90^	18	NA	18^a^	18	NA	NA	NA	NA
Philippines^92^^,^^98^	3	3	3	3	3	3	3	3
Sri Lanka^99^^–^^103^^,^^105^^,^^154^	NA	NA	Once	NA	Once	NA	NA	NA
Thailand^107^^,^^108^^,^^162^	NA	NA	NA	NA	NA	NA	NA	NA
Timor-Leste^112^^,^^114^	Once	NA	Once^a^	Once	NA	Once	NA	NA
Viet Nam^116^^,^^117^^,^^120^^,^^163^	5^b^	NA	5^a,b,d^	5^b,d^	5^b,d^	5^b,d^	NA	NA
**Vitamin D (plasma or serum levels)**								
Bangladesh^22^^,^^24^^,^^26^^,^^157^	8	Once	Once	8	NA	NA	NA	NA
Bhutan^27^^–^^29^	NA	NA	NA	NA	NA	NA	NA	NA
Brunei Darussalam^31^^–^^34^	NA	NA	NA	NA	NA	NA	NA	NA
Cambodia^36^	Once^e^	NA	Once^a,e^	Once^e^	Once^e^	Once^e^	NA	NA
China^37^^,^^38^^,^^40^^,^^49^	3	3	3	3	3	3	3	3
Democratic People's Republic of Korea^50^	NA	NA	NA	NA	NA	NA	NA	NA
India^52^^,^^53^^,^^55^^–^^64^	Once	Once	3,^a,b^ once	Once	Once	Once	Once	NA
Indonesia^67^^,^^68^^,^^71^	NA	NA	NA	NA	NA	NA	NA	NA
Lao People's Democratic Republic^47^^,^^72^^,^^73^	NA	NA	NA	NA	NA	NA	NA	NA
Malaysia^48^^,^^78^^,^^79^	NA	NA	NA	NA	NA	NA	NA	NA
Maldives^80^^,^^81^	NA	NA	NA	NA	NA	NA	NA	NA
Myanmar^83^^–^^85^	Once	NA	Once^a^	Once	Once	Once	NA	NA
Nepal^87^^–^^90^	NA	NA	NA	NA	NA	NA	NA	NA
Philippines^92^^,^^98^	3	3	3	3	3	3	3	3
Sri Lanka^99^^–^^103^^,^^105^^,^^154^	NA	NA	One round	NA	NA	NA	NA	NA
Thailand^107^^,^^108^^,^^162^	NA	NA	NA	NA	NA	NA	NA	NA
Timor-Leste^112^^,^^114^	NA	NA	NA	NA	NA	NA	NA	NA
Viet Nam^116^^,^^117^^,^^120^^,^^163^	NA	NA	NA	NA	NA	NA	NA	NA
**Vitamin B12**								
Bangladesh^22^^,^^24^^,^^26^^,^^157^	NA	NA	8^a^	8	NA	NA	NA	NA
Bhutan^27^^–^^29^	NA	NA	NA	NA	NA	NA	NA	NA
Brunei Darussalam^31^^–^^34^	NA	NA	NA	NA	NA	NA	NA	NA
Cambodia^36^	Once^e^	NA	Once^a,e^	Once^e^	Once^e^	Once^e^	NA	NA
China^37^^,^^38^^,^^40^^,^^49^	3	NA	NA	NA	NA	3	NA	NA
Democratic People's Republic of Korea^50^	NA	NA	NA	NA	NA	NA	NA	NA
India^52^^,^^53^^,^^55^^–^^64^	Once	Once	Once	NA	NA	NA	NA	NA
Indonesia^67^^,^^68^^,^^71^	NA	NA	NA	NA	NA	NA	NA	NA
Lao People's Democratic Republic^47^^,^^72^^,^^73^	NA	NA	NA	NA	NA	NA	NA	NA
Malaysia^48^^,^^78^^,^^79^	NA	NA	NA	NA	NA	NA	NA	NA
Maldives^80^^,^^81^	NA	NA	NA	NA	NA	NA	NA	NA
Myanmar^83^^–^^85^	NA	NA	NA	NA	NA	NA	NA	NA
Nepal^87^^–^^90^	NA	NA	NA	NA	NA	NA	NA	NA
Philippines^92^^,^^98^	NA	NA	NA	NA	NA	NA	NA	NA
Sri Lanka^99^^–^^103^^,^^105^^,^^154^	NA	NA	NA	NA	NA	NA	NA	NA
Thailand^107^^,^^108^^,^^162^	NA	NA	NA	NA	NA	NA	NA	NA
Timor-Leste^112^^,^^114^	NA	NA	NA	NA	NA	NA	NA	NA
Viet Nam^116^^,^^117^^,^^120^^,^^163^	10	NA	10	NA	10	10	NA	NA
**Vitamin B1 (thiamine levels)**								
Bangladesh^22^^,^^24^^,^^26^^,^^157^	NA	NA	NA	NA	NA	NA	NA	NA
Bhutan^27^^–^^29^	NA	NA	NA	NA	NA	NA	NA	NA
Brunei Darussalam^31^^–^^34^	NA	NA	NA	NA	NA	NA	NA	NA
Cambodia^36^	NA	NA	NA	NA	NA	NA	NA	NA
China^37^^,^^38^^,^^40^^,^^49^	NA	NA	NA	NA	NA	3	NA	NA
Democratic People's Republic of Korea^50^	NA	NA	NA	NA	NA	NA	NA	NA
India^52^^,^^53^^,^^55^^–^^64^	NA	NA	NA	NA	NA	NA	NA	NA
Indonesia^67^^,^^68^^,^^71^	NA	NA	NA	NA	NA	NA	NA	NA
Lao People's Democratic Republic^47^^,^^72^^,^^73^	NA	NA	NA	NA	NA	NA	NA	NA
Malaysia^48^^,^^78^^,^^79^	NA	NA	NA	NA	NA	NA	NA	NA
Maldives^80^^,^^81^	NA	NA	NA	NA	NA	NA	NA	NA
Myanmar^83^^–^^85^	NA	NA	Once^a^	Once	Once	Once	NA	NA
Nepal^87^^–^^90^	NA	NA	NA	NA	NA	NA	NA	NA
Philippines^92^^,^^98^	NA	NA	NA	NA	NA	NA	NA	NA
Sri Lanka^99^^–^^103^^,^^105^^,^^154^	NA	NA	NA	NA	NA	NA	NA	NA
Thailand^107^^,^^108^^,^^162^	NA	NA	NA	NA	NA	NA	NA	NA
Timor-Leste^112^^,^^114^	NA	NA	NA	NA	NA	NA	NA	NA
Viet Nam^116^^,^^117^^,^^120^^,^^163^	10	NA	10^a^	10	NA	10	NA	NA
**Calcium**								
Bangladesh^22^^,^^24^^,^^26^^,^^157^	Once	Once	Once	Once	NA	NA	NA	NA
Bhutan^27^^–^^29^	NA	NA	NA	NA	NA	NA	NA	NA
Brunei Darussalam^31^^–^^34^	NA	NA	NA	NA	NA	NA	NA	NA
Cambodia^36^	Once^e^	NA	Once^a,e^	Once^e^	Once^e^	Once^e^	NA	NA
China^37^^,^^38^^,^^40^^,^^49^	NA	NA	NA	NA	NA	NA	NA	NA
Democratic People's Republic of Korea^50^	NA	NA	NA	NA	NA	NA	NA	NA
India^52^^,^^53^^,^^55^^–^^64^	NA	NA	NA	NA	NA	NA	NA	NA
Indonesia^67^^,^^68^^,^^71^	NA	NA	NA	NA	NA	NA	NA	NA
Lao People's Democratic Republic^47^^,^^72^^,^^73^	NA	NA	NA	NA	NA	NA	NA	NA
Malaysia^48^^,^^78^^,^^79^	NA	NA	NA	NA	NA	NA	NA	NA
Maldives^80^^,^^81^	NA	NA	NA	NA	NA	NA	NA	NA
Myanmar^83^^–^^85^	NA	NA	NA	NA	NA	NA	NA	NA
Nepal^87^^–^^90^	NA	NA	NA	NA	NA	NA	NA	NA
Philippines^92^^,^^98^	NA	NA	NA	NA	NA	NA	NA	NA
Sri Lanka^99^^–^^103^^,^^105^^,^^154^	Once	NA	NA	NA	NA	NA	NA	NA
Thailand^107^^,^^108^^,^^162^	NA	NA	NA	NA	NA	NA	NA	NA
Timor-Leste^112^^,^^114^	NA	NA	NA	NA	NA	NA	NA	NA
Viet Nam^116^^,^^117^^,^^120^^,^^163^	10	NA	10	10	10	10	NA	NA
**Folate (Vitamin B9: red blood cell or serum folate)**								
Bangladesh^22^^,^^24^^,^^26^^,^^157^	NA	NA	8^a^	8	NA	NA	NA	NA
Bhutan^27^^–^^29^	NA	NA	NA	NA	NA	NA	NA	NA
Brunei Darussalam^31^^–^^34^	NA	NA	NA	NA	NA	NA	NA	NA
Cambodia^36^	Once^e ^	NA	Once^a,e^	Once^e^	Once^e^	Once^e^	NA	NA
China^37^^,^^38^^,^^40^^,^^49^	3	NA	NA	NA	NA	3	NA	NA
Democratic People's Republic of Korea^50^	NA	NA	NA	NA	NA	NA	NA	NA
India^52^^,^^53^^,^^55^^–^^64^	Once	Once	Once	NA	NA	NA	NA	NA
Indonesia^67^^,^^68^^,^^71^	NA	NA	NA	NA	NA	NA	NA	NA
Lao People's Democratic Republic ^47^^,^^72^^,^^73^	NA	NA	NA	NA	NA	NA	NA	NA
Malaysia^48^^,^^78^^,^^79^	NA	NA	NA	NA	NA	NA	NA	NA
Maldives^80^^,^^81^	NA	NA	NA	NA	NA	NA	NA	NA
Myanmar^83^^–^^85^	NA	NA	On round^a^	Once	Once	Once	NA	NA
Nepal^87^^–^^90^	Once	Once	Once	Once	NA	Once	NA	NA
Philippines^92^^,^^98^	3	3	3	3	3	3	3	3
Sri Lanka^99^^–^^103^^,^^105^^,^^154^	NA	NA	NA	NA	NA	NA	NA	NA
Thailand^107^^,^^108^^,^^162^	NA	NA	NA	NA	NA	NA	NA	NA
Timor-Leste^112^^,^^114^	NA	NA	NA	NA	NA	NA	NA	NA
Viet Nam^116^^,^^117^^,^^120^^,^^163^	10	NA	10^a^	10	10	10	NA	NA
**Iron (serum ferritin or soluble transferrin receptor)**								
Bangladesh^22^^,^^24^^,^^26^^,^^157^	8	Once	Once	8	NA	NA	NA	NA
Bhutan^27^^–^^29^	NA	NA	NA	NA	NA	NA	NA	NA
Brunei Darussalam^31^^–^^34^	NA	NA	NA	NA	NA	NA	NA	NA
Cambodia^36^	Once^e^	NA	Once^a,e^	Once^e^	Once^e^	Once^e^	NA	NA
China^37^^,^^38^^,^^40^^,^^49^	3^b^	3^b^	3^b^	3^b^	3^b^	3^b^	3^b^	3^b^
Democratic People's Republic of Korea^50^	NA	NA	NA	NA	NA	NA	NA	NA
India^52^^,^^53^^,^^55^^–^^64^	Once	Once	Once	NA	NA	NA	NA	NA
Indonesia^67^^,^^68^^,^^71^	NA	NA	NA	NA	NA	NA	NA	NA
Lao People's Democratic Republic^47^^,^^72^^,^^73^	NA	NA	NA	NA	NA	NA	NA	NA
Malaysia^48^^,^^78^^,^^79^	NA	NA	NA	NA	NA	NA	NA	NA
Maldives^80^^,^^81^	NA	NA	NA	NA	NA	NA	NA	NA
Myanmar^83^^–^^85^	Once	NA	Once^a^	Once	Once	Once	NA	NA
Nepal^87^^–^^90^	NA	NA	NA	NA	NA	NA	NA	NA
Philippines^92^^,^^98^	NA	NA	NA	NA	NA	NA	NA	NA
Sri Lanka^99^^–^^103^^,^^105^^,^^154^	Once	NA	Once	NA	Once	NA	NA	NA
Thailand^107^^,^^108^^,^^162^	NA	NA	NA	NA	NA	NA	NA	NA
Timor-Leste^112^^,^^114^	Once	NA	NA	Once	NA	Once	NA	NA
Viet Nam^116^^,^^117^^,^^120^^,^^163^	5^b,d^	NA	10	5^b,d^	5^b,d^	5^b,d^	NA	NA
**Zinc**								
Bangladesh^22^^,^^24^^,^^26^^,^^157^	8	NA	8^a^	8	NA	NA	NA	NA
Bhutan^27^^–^^29^	NA	NA	NA	NA	NA	NA	NA	NA
Brunei Darussalam^31^^–^^34^	NA	NA	NA	NA	NA	NA	NA	NA
Cambodia^36^	Once^e^	NA	Once^a,e^	Once^e^	Once^e^	Once^e^	NA	NA
China^37^^,^^38^^,^^40^^,^^49^	3	3	3	3	3	3	3	3
Democratic People's Republic of Korea^50^	NA	NA	NA	NA	NA	NA	NA	NA
India^52^^,^^53^^,^^55^^–^^64^	Once	Once	Once	NA	NA	NA	NA	NA
Indonesia^67^^,^^68^^,^^71^	NA	NA	NA	NA	NA	NA	NA	NA
Lao People's Democratic Republic^47^^,^^72^^,^^73^	NA	NA	NA	NA	NA	NA	NA	NA
Malaysia^48^^,^^78^^,^^79^	NA	NA	NA	NA	NA	NA	NA	NA
Maldives^80^^,^^81^	NA	NA	NA	NA	NA	NA	NA	NA
Myanmar^83^^–^^85^	Once	NA	Once^c^	NA	NA	NA	NA	NA
Nepal^87^^–^^90^	Once	NA	Once^a^	Once	Once	Once	NA	NA
Philippines^92^^,^^98^	NA	NA	NA	NA	NA	NA	NA	NA
Sri Lanka^99^^–^^103^^,^^105^^,^^154^	Once	NA	Once	NA	NA	NA	NA	NA
Thailand^107^^,^^108^^,^^162^	NA	NA	NA	NA	NA	NA	NA	NA
Timor-Leste^112^^,^^114^	Once	NA	Once^a^	Once	NA	Once	NA	NA
Viet Nam^116^^,^^117^^,^^120^^,^^163^	5^b,d^	NA	5^a,b,d^	5^b,d^	5^b,d^	5^b,d^	NA	NA
**Urinary iodine**								
Bangladesh^22^^,^^24^^,^^26^^,^^157^	Once	Once	Once, 8^a^	8	NA	NA	NA	NA
Bhutan^27^^–^^29^	NA	NA	NA	NA	NA	NA	NA	NA
Brunei Darussalam^31^^–^^34^	NA	NA	NA	NA	NA	NA	NA	NA
Cambodia^36^	Once^e ^	NA	Once^a,e^	Once^e^	Once^e^	Once^e^	NA	NA
China^37^^,^^38^^,^^40^^,^^49^	NA	NA	NA	NA	NA	Once	NA	NA
Democratic People's Republic of Korea^50^	NA	NA	NA	NA	NA	NA	NA	NA
India^52^^,^^53^^,^^55^^–^^64^	Once	Once	Once	NA	NA	NA	NA	NA
Indonesia^67^^,^^68^^,^^71^	NA	NA	NA	NA	NA	NA	NA	NA
Lao People's Democratic Republic^47^^,^^72^^,^^73^	NA	NA	NA	NA	NA	NA	NA	NA
Malaysia^48^^,^^78^^,^^79^	NA	Once	NA	NA	Routine surveillance	NA	NA	NA
Maldives^80^^,^^81^	NA	NA	NA	NA	NA	NA	NA	NA
Myanmar^83^^–^^85^	NA	Once	NA	NA	Once	NA	NA	NA
Nepal^87^^–^^90^	NA	18	18^a^	18	NA	Once	NA	NA
Philippines^92^^,^^98^	3	3	3	3	3	3	3	3
Sri Lanka^99^^–^^103^^,^^105^^,^^154^	NA	4–6	Once	NA	Once	NA	NA	NA
Thailand^107^^,^^108^^,^^162^	NA	NA	NA	5–6	NA	Annually	5–6	NA
Timor-Leste^112^^,^^114^	NA	NA	NA	Once	NA	Once	NA	NA
Viet Nam^116^^,^^117^^,^^120^^,^^163^	10	NA	10	10	10	10	NA	NA
**Noncommunicable disease biomarkers**								
**CRP or acid glycoprotein**								
Bangladesh^22^^,^^24^^,^^26^^,^^157^	NA	Once	Once	Once	NA	NA	NA	NA
Bhutan^27^^–^^29^	NA	NA	NA	NA	NA	NA	NA	NA
Brunei Darussalam ^31^^–^^34^	NA	NA	NA	NA	NA	NA	NA	NA
Cambodia^36^	NA	NA	NA	NA	NA	NA	NA	NA
China^37^^,^^38^^,^^40^^,^^49^	NA	3	3	3	3	3	3	3
Democratic People's Republic of Korea^50^	NA	NA	NA	NA	NA	NA	NA	NA
India^52^^,^^53^^,^^55^^–^^64^	NA	Once	Once	NA	NA	NA	NA	NA
Indonesia^67^^,^^68^^,^^71^	NA	NA	NA	NA	NA	NA	NA	NA
Lao People's Democratic Republic^47^^,^^72^^,^^73^	NA	NA	NA	NA	NA	NA	NA	NA
Malaysia^48^^,^^78^^,^^79^	NA	NA	NA	NA	NA	NA	NA	NA
Maldives^80^^,^^81^	NA	NA	NA	NA	NA	NA	NA	NA
Myanmar^83^^–^^85^	NA	Once	Once	Once	Once	Once	NA	NA
Nepal^87^^–^^90^	Once	NA	Once	Once	Once	NA	NA	NA
Philippines^92^^,^^98^	NA	NA	NA	NA	NA	NA	NA	NA
Sri Lanka^99^^–^^103^^,^^105^^,^^154^	Once	NA	Once	NA	Once	NA	NA	NA
Thailand^107^^,^^108^^,^^162^	NA	NA	NA	NA	NA	NA	NA	NA
Timor-Leste^112^^,^^114^	NA	NA	NA	NA	NA	NA	NA	NA
Viet Nam^116^^,^^117^^,^^120^^,^^163^	NA	NA	NA	NA	NA	NA	NA	NA
**Fasting blood glucose**								
Bangladesh^22^^,^^24^^,^^26^^,^^157^	NA	NA	NA	1–4^b^	1–4^b^	3–6	3–6	NA
Bhutan^27^^–^^29^	NA	NA	5–7^a^	5–7	5–7	NA	5–7	NA
Brunei Darussalam Darussalam^31^^–^^34^	NA	NA	NA	5–6^b^	NA	NA	12	12
Cambodia^36^	NA	NA	NA	NA	NA	NA	NA	NA
China^37^^,^^38^^,^^40^^,^^49^	NA	3^b^	3^b^	1–3^b^	3^b^	3	1–3^b^	1–3^b^
Democratic People's Republic of Korea^50^	NA	NA	NA	NA	NA	NA	NA	NA
India^52^^,^^53^^,^^55^^–^^64^	Once	Once	Once	2^b^	2^b^	4^b^	2^b^	Once
Indonesia^67^^,^^68^^,^^71^	NA	NA	NA	3–5	3–5	3–5	3–5	3–5
Lao People's Democratic Republic^47^^,^^72^^,^^73^	NA	NA	NA	Once	Once	NA	Once	NA
Malaysia^48^^,^^78^^,^^79^	NA	NA	NA	4–5	4–5	NA	4–5	4–5
Maldives^80^^,^^81^	NA	NA	7–11^a^	7–11	7–11	NA	7–11	NA
Myanmar^83^^–^^85^	NA	NA	NA	4–5	4–5	NA	4–5	4–5
Nepal^87^^–^^90^	NA	NA	2–6^a,b^	2–6^b^	2–6^b^	Once	2–6^b^	NA
Philippines^92^^,^^98^	NA	NA	3^a^	3	3	3	3	3
Sri Lanka^99^^–^^103^^,^^105^^,^^154^	NA	NA	NA	Once	Once	NA	Once	NA
Thailand^107^^,^^108^^,^^162^	NA	NA	NA	3^b^	NA	NA	3^b^	NA
Timor-Leste^112^^,^^114^	NA	NA	NA	Once	Once	NA	Once	NA
Viet Nam^116^^,^^117^^,^^120^^,^^163^	NA	NA	NA	3	3	3	3	NA
**Cholesterol level**								
Bangladesh^22^^,^^24^^,^^26^^,^^157^	NA	NA	NA	Once	Once	NA	NA	NA
Bhutan^27^^–^^29^	NA	NA	NA	5–7	5–7	NA	5–7	NA
Brunei Darussalam^31^^–^^34^	NA	NA	NA	5–6^b^	NA	NA	5–6^b^	12
Cambodia^36^	NA	NA	NA	NA	NA	NA	NA	NA
China^37^^,^^38^^,^^40^^,^^49^	NA	3^b^	3^b^	1–3^b^	NA	NA	1–3^b^	1–3^b^
Democratic People's Republic of Korea^50^	NA	NA	NA	NA	NA	NA	NA	NA
India^52^^,^^53^^,^^55^^–^^64^	NA	Once	Once	Once (urban)	NA	NA	Once (urban)	NA
Indonesia^67^^,^^68^^,^^71^	NA	NA	NA	3–5	3–5	3–5	3–5	3–5
Lao People's Democratic Republic^47^^,^^72^^,^^73^	NA	NA	NA	Once	Once	NA	Once	NA
Malaysia^48^^,^^78^^,^^79^	NA	NA	NA	4–5	4–5	4–5	4–5	4–5
Maldives^80^^,^^81^	NA	NA	7–11^a^	7–11	7–11	NA	7–11	NA
Myanmar^83^^–^^85^	NA	NA	NA	Once	Once	NA	Once	NA
Nepal^87^^–^^90^	NA	NA	6^a^	6	6	NA	6	NA
Philippines^92^^,^^98^	NA	NA	NA	NA	NA	NA	NA	NA
Sri Lanka^99^^–^^103^^,^^105^^,^^154^	NA	NA	NA	Once	Once	NA	Once	NA
Thailand^107^^,^^108^^,^^162^	NA	NA	NA	6	NA	NA	6	NA
Timor-Leste^112^^,^^114^	NA	NA	NA	Once	Once	NA	Once	NA
Viet Nam^116^^,^^117^^,^^120^^,^^163^	NA	NA	NA	6	6	NA	6	NA

CRP: C-reactive protein; Hb: Haemoglobin; NA: not applicable.

^a^ Only covers adolescents 15–19 years of age.

^b^ Monitoring by multiple programmes.

^c ^Only covers younger adolescents 10–14 years of age.

^d ^Micronutrient Survey 2014–15. This survey may see a new data collection round.

^e^ One-time micronutrient survey together with 2014 Demographic and Health Survey.^142^

Note: No information collected on selenium among countries.

Dietary intake monitoring also varied substantially across countries (Table 3 and Table 4). Periodic collection of individual food intake data occurred in all countries, of which 17 out of 18 countries are using food frequency questionnaires in varying lengths and with a focus on different aspects. These questionnaires mainly focus on specific behaviours linked to diet-related noncommunicable diseases. Most countries (Bangladesh, Brunei Darussalam, Cambodia, China, Democratic People's Republic of Korea, India, Lao People's Democratic Republic, Malaysia, Nepal, Philippines, Sri Lanka, Thailand, Timor-Leste and Viet Nam) also use a 24-hour dietary recall method in one, or multiple, of their programmes to measure individual intake.^33^^,^^36^^,^^38^^,^^50^^,^^58^^,^^59^^,^^78^^,^^87^^,^^89^^,^^92^^,^^111^^,^^116^^,^^135^^,^^142^^,^^156^ China uses weighed food records to measure quantitative information on individual diets.^38^ All countries except Brunei Darussalam periodically collect information on infant and young child feeding practices, with an interval between 1 to 10 years.

**Table 3:** Individual dietary assessment in study countries

Measurement, by country	Interval between rounds, years, by target group							
	Children < 5 years	Children 5–9 years	Adolescents 10–19 years	Women of reproductive age	Pregnant women	Lactating women	Adult men	Elderly people
**Individual dietary assessment**								
**Food frequency questionnaire** ^a^								
Bangladesh^22^^,^^24^^,^^26^^,^^157^	NA	NA	Once^b^	3–5^c^	3–5^c^	3–5^c^	3–5^c^	NA
Bhutan^27^^–^^29^	NA	NA	2–5,^c,d,e^ once^b^	2–5^c,e^	2–5^c,e^	NA	2–5^c,e^	2–5^c,e^
Brunei Darussalam^31^^–^^34^	NA	10^c^	12, 5^b^	4–6^c,e^	4–6^c,e^	12	4–6^c,e^	NA
Cambodia^36^	NA	NA	Once^b^	NA	NA	NA	NA	NA
China^37^^,^^38^^,^^40^^,^^49^	2^b^	NA	3,^e^ 2^d,e^	2^e^	3^e^	3	2^e^	2^e^
Democratic People's Republic of Korea^50^	NA	NA	NA	NA	NA	NA	NA	NA
India^52^^,^^53^^,^^55^^–^^64^	NA	NA	4–10,^d^ once^c^	4–10, once^c^	4–10, once^c^	4–10	4–10, once^c^	Once
Indonesia^67^^,^^68^^,^^71^	3–5^c^	3–5^c^	3–5,^c^ once^d^	3–5^c^	3–5^c^	3–5^c^	3–5^c^	3–5^c^
Lao People's Democratic Republic^47^^,^^72^^,^^73^	NA	NA	7^b^	5^c^	5^c^	NA	5^c^	NA
Malaysia^48^^,^^78^^,^^79^	NA	NA	5	11, 4–5^c^	4–5^c^	4–5^c^	11, 4–5^c^	Once
Maldives^80^^,^^81^	NA	NA	7–11,^d^ 5^b^	7–11^c^	7–11^c^	NA	7–11^c^	NA
Myanmar^83^^–^^85^	NA	NA	8^b^	4–5^c^	4–5^c^	NA	4–5^c^	NA
Nepal^87^^–^^90^	NA	NA	2–6,^d^ once^b^	2–6^c^	2–6^c^	NA	2–6^c^	NA
Philippines^109,161^	NA	NA	4^b^	NA	NA	NA	NA	NA
Sri Lanka^99^^–^^103^^,^^105^^,^^154^	NA	Once	Once, 4–7,^d^ 8^b^	4–7^c^	Once	Once	4–7^c^	NA
Thailand^107^^,^^108^^,^^162^	5–6^e^	5–6,^e^ once^c^	5–6,^e^ once^c^	5–6, 3^c^	3^c^	NA	5–6,^e^ 3^c^	5–6, 3^c^
Timor-Leste^112^^,^^114^	NA	NA	7^b^	Once^c^	Once^c^	NA	Once^c^	NA
Viet Nam^116^^,^^117^^,^^120^^,^^163^	NA	NA	6^b^	6^c^	6^c^	NA	6^c^	NA
**24-hour recall**								
Bangladesh^22^^,^^24^^,^^26^^,^^157^	3^f^	NA	Once^d^, 3^f^	3^f^	NA	NA	Once^f^	Once^f^
Bhutan^27^^–^^29^	NA	NA	NA	NA	NA	NA	NA	NA
Brunei Darussalam^31^^–^^34^	NA	12	12	12	NA	12	12	12
Cambodia^36^	NA	NA	Once^d,f^	Once^f^	Once^f^	Once^f^	NA	NA
China^37^^,^^38^^,^^40^^,^^49^	NA	3	3	3	3	3	3	3
Democratic People's Republic of Korea^50^	NA	NA	Once^d^	Once	Once	Once	NA	NA
India^52^^,^^53^^,^^55^^–^^64^	Once^e,f^	Once^f^	Once^f^	NA	NA	NA	NA	NA
Indonesia^67^^,^^68^^,^^71^	NA	NA	NA	NA	NA	NA	NA	NA
Lao People's Democratic Republic^47^^,^^72^^,^^73^	NA	NA	NA	Biannually	NA	NA	NA	NA
Malaysia^48^^,^^78^^,^^79^	NA	NA	5	11	NA	NA	11	NA
Maldives^80^^,^^81^	NA	NA	NA	NA	NA	NA	NA	NA
Myanmar^83^^–^^85^	NA	NA	NA	NA	NA	NA	NA	NA
Nepal^87^^–^^90^	NA	Once^e,f^	6,^d,e^ once^f^	6^e,f^	6^e,f^	6^e,f^	NA	NA
Philippines^92^^,^^98^	3	3	3, once^d,f^	3, once^f^	3, once^f^	3, once^f^	3	3
Sri Lanka^99^^–^^103^^,^^105^^,^^154^	Once	NA	Once^f^	10^f^	10^f^	10^f^	NA	NA
Thailand^107^^,^^108^^,^^162^	10	10	10	10	10	10	10	10
Timor-Leste^112^^,^^114^	NA	NA	Once^f^	Once^f^	Once^f^	Once^f^	NA	NA
Viet Nam^116^^,^^117^^,^^120^^,^^163^	NA	NA	Once	Once	Once	Once	Once	NA
**Other**								
Bangladesh^22^^,^^24^^,^^26^^,^^157^	NA	NA	Once^g^ once^d,h^	5^h^	5^h^	NA	NA	NA
Bhutan^27^^–^^29^	NA	NA	Once^g^	NA	NA	NA	NA	NA
Brunei Darussalam^31^^–^^34^	NA	NA	Once^g^	Once^g^	Once^g^	Once^g^	Once^g^	Once^g^
Cambodia^36^	NA	NA	Once^g^	NA	4–5^h^	NA	NA	NA
China^37^^,^^38^^,^^40^^,^^49^	NA	NA	NA	NA	NA	NA	NA	NA
Democratic People's Republic of Korea^50^	NA	NA	NA	NA	NA	NA	NA	NA
India^52^^,^^53^^,^^55^^–^^64^	NA	NA	NA	NA	NA	NA	NA	NA
Indonesia^67^^,^^68^^,^^71^	NA	NA	8^g^	NA	1^e,h^	1^e,h^	NA	NA
Lao People's Democratic Republic^47^^,^^72^^,^^73^	NA	NA	7^g^	NA	NA	NA	NA	NA
Malaysia^48^^,^^78^^,^^79^	NA	Once^h^	5^h^	10^h^ once^i^	Once^i^	NA	Once,^h^ once^h^	Once^i^
Maldives^80^^,^^81^	NA	NA	5^g^	NA	7^h^	NA	NA	NA
Myanmar^83^^–^^85^	NA	NA	8^g^	NA	NA	NA	NA	NA
Nepal^87^^–^^90^	NA	NA	Once^g^, once^h^	Once^h^	Once^h^	Once^h^	NA	NA
Philippines^92^^,^^98^	NA	NA	4^g^	NA	4–5^h^	NA	NA	NA
Sri Lanka^99^^–^^103^^,^^105^^,^^154^	NA	NA	8^h^	NA	Once^h^	NA	NA	NA
Thailand^107^^,^^108^^,^^162^	NA	NA	7^g^	NA	NA	NA	NA	NA
Timor-Leste^112^^,^^114^	NA	NA	Once^g^	7^g^	7^g^	7^g^	NA	NA
Viet Nam^116^^,^^117^^,^^120^^,^^163^	NA	NA	6,^g^ once^h^	One round^h^	Once^h^	Once^h^	NA	NA

NA: Not applicable.

^a^ Includes short versions adapted to different survey platforms (World Health Organization STEPwise approach)^164^ that differ per programme and country, often specific foods associated with increased noncommunicable disease risk.

^b^ Food frequency questionnaire in the Global School-based Student Health Survey.^165^

^c^ Food frequency questionnaire in the World Health Organization STEPwise approach to noncommunicable disease risk factor surveillance.^164^

^d^ Only covers adolescents 15–19 years of age.

^e^ Monitored by multiple programmes.

^f^ 24-hour recall questionnaire.

^g^ Individual food insecurity question(s) on lack of food over a specific period of time.

^h^ Individual micronutrient intake through supplementation and/or specific micronutrient-dense foods.

^i^ Three questions on commercially packed ready to drink beverages.

**Table 4:** Household-level dietary assessments in study countries

Country	Interval between rounds of assessment, years						
	Household level						Infant and young child feeding practices
	Iodized salt intake	24-hour food recall	Food insecurity access scale	Food frequency questionnaire	Food insecurity experience scale	Other	
Bangladesh^20^^,^^22^^,^^24^^,^^26^^,^^157^	8	5	1^a^	NA	NA	Once^b^	6^a^
Bhutan^27^^–^^29^	NA	NA	NA	NA	7	NA	7
Brunei Darussalam ^31^^–^^34^	NA	NA	NA	NA	NA	NA	NA
Cambodia^36^^,^^145^	NA	NA	NA	2	NA	2^c^	2–5^a^
China^37^^,^^38^^,^^40^^,^^49^	NA	2–4	NA	NA	NA	3^d^	1
Democratic People's Republic of Korea^50^^,^^51^	8	NA	NA	NA	NA	NA	3–5^a^
India^52^^,^^53^^,^^55^^–^^64^	NA	Once	NA	Once	NA	NA	2–4^a^
Indonesia^67^^,^^68^^,^^71^^,^^129^^,^^130^	NA	NA	NA	Annually	Annually^a^	NA	1^a^
Lao People's Democratic Republic ^47^^,^^72^^,^^73^	5	NA	NA	NA	NA	NA	5
Malaysia^48^^,^^76^^,^^78^^,^^79^^,^^151^	Four-year cycles from 2024	NA	NA	NA	11	NA	5^a^
Maldives^80^^,^^81^^,^^95^	NA	NA	NA	NA	NA	NA	5
Myanmar^83^^–^^85^	Once	Once	Once	NA	NA	NA	2^a^
Nepal^87^^–^^90^^,^^152^^,^^153^	Once	NA	5^a^	NA	NA	Once^b^	2–3^a^
Philippines^92^^,^^93^^,^^98^	3	NA	3	NA	NA	3^e^	3^a^
Sri Lanka^99^^–^^103^^,^^104^^,^^105^^,^^154^	4–6^a^	NA	NA	NA	NA	NA	10
Thailand^107^^,^^108^^,^^109^^,^^162^	5–7	NA	NA	NA	NA	NA	5–7
Timor-Leste^111^^,^^112^^,^^114^	7	NA	7	NA	NA	NA	3–4^a^
Viet Nam^116^^–^^121^^,^^163^	NA	10	NA	7	NA	10^f^	3^a^

NA: not applicable.

^a^ Monitored by multiple programmes.

^b^ Household use and purchase of fortified food.

^c^ Household food-related coping strategies.

^d^ Food records assessment of individuals living in households with children who are younger than 5 years.

^e^ Household one-day food weighing as part of 24-hour recall.

^f^ Household food security (experienced a food shortage during the last year).

In Bangladesh, Sri Lanka and Thailand, iodine content is measured in salt production as part of their national nutrition survey. Generally there is limited nutrition-related food environment and food system monitoring within the identified surveillance programmes. Moreover, we did not identify any government-led programmes run by non-health departments that included food environment and/or wider food system indicators, or monitored data across the food system with the purpose of controlling malnutrition.

### Data quality

To ensure high quality of the collected data, all countries use rigorous supervision, personnel training and applying the most recently available global standards at the time of programme implementation. A total of 49 national and internationally linked programmes report higher than 80% response rates (range: 53–99). Several individual programmes in Brunei Darussalam, India, Maldives and Sri Lanka had lower-than targeted response rates due to respondents’ poor access to field sites or refusal of biochemical measurements.^32^^,^^33^^,^^35^^,^^52^^,^^58^^,^^80^^–^^82^^,^^105^ National or local country surveillance programmes showed evidence of flexibility as they expanded indicators and/or subpopulation groups between their latest data collection rounds.

### Representativeness

Coverage of population groups for nutrition-outcome indicators differs to a large extent. Except Brunei Darussalam, all countries cover anthropometric information on nutrition status among children younger than 5 years, and all countries include women of reproductive age for anthropometry at varying time intervals.

Bangladesh, China, India, Indonesia, Malaysia, Philippines and Thailand have national surveillance that covers measurement of anthropometric nutrition status among all age groups (Table 1). Common missing groups across most countries’ surveillance were elderly people for anthropometry, and school-aged children and elderly people for dietary assessment.

All countries collect nutrition and diet outcome data that can be disaggregated by key sociodemographic factors, including socioeconomic status. Six countries rely on the Global School-Based Student Health Surveys for young adolescent (10–14 years of age) weight and height (self-reported) which cannot be disaggregated by socioeconomic status. Aside from one programme in Bangladesh and two in India, urban deprived areas or informal settlements, and mobile populations (including homeless, internally displaced people, refugees, nomadic populations) are generally not represented within national nutrition surveillance.

### Timeliness and simplicity

All countries have programmes that are digitized, mainly through the incorporation of computer-assisted personal interviews (that is, a face-to-face data collection method in which the interviewer uses a tablet, mobile phone or a computer to record answers given during the interview) in their most recent surveillance rounds. While there is limited annual overlap between identified programmes for collected nutrition data within countries, data for infant and young child feeding were overlapping in the Philippines and Viet Nam; ^92^^,^^93^^,^^98^^,^^116^^,^^118^^–^^121^ and adult weight and height were collected among different samples by separate programmes in China and Viet Nam.^37^^–^^49^^,^^116^^–^^121^

### Stability

Included programmes in most countries did not report to have experienced any preparation or operation issues in their latest round. Reported issues mainly related to financial costs (two programmes), logistical challenges (two programmes), and few trained data collection personnel (one programme). Most countries’ continuous and periodic programmes collected data with consistent time intervals between rounds and with limited interruptions. Bangladesh, China, India, Indonesia, Malaysia, Philippines, Sri Lanka, Thailand and Viet Nam have exclusive nutrition-focused programmes that were fully funded by their respective national governments.^21^^,^^38^^,^^40^^–^^43^^,^^46^^,^^49^^,^^52^^,^^59^^,^^78^^,^^93^^,^^95^^–^^97^^,^^104^^,^^121^^,^^151^^,^^156^ Other countries’ national nutrition surveillance mainly includes programmes that were reliant on external support.

## Discussion

Through our analysis of publicly available literature and consultations with national nutrition and health officials, we identified and described ongoing national and internationally linked nutrition surveillance programmes for 18 countries. Our review shows large variations between countries in terms of scope, and frequency of monitoring. Many countries implement one or multiple nutrition- and diet-focused periodical surveillance programmes with wide intervals. Few countries collect continuous comprehensive information on individual diet and micronutrient biomarkers. The latter finding is consistent with a recent review on the availability and use of micronutrient data in low- and middle-income countries worldwide.^166^ While individual dietary data and biochemical measures of micronutrient status are highly accurate, continuous collection of such data is time- and cost-intensive.^167^ More recent innovative dietary assessments – for example, the diet quality questionnaire by the Global Diet Quality Project – have been developed and trialled in some south-east Asian countries, which can help reduce cost and participant burden.^168^

While the identified nutrition surveillance programmes generally allow for disaggregation of important nutrition-related sociodemographic variables, most surveys do not accurately represent populations in vulnerable settings with prevalent malnutrition issues. Many low- and middle-income countries have a substantial proportion of their population living in such settings,^169^ hence we recommend scaling up national surveillance programmes to go beyond sentinel surveillance in these settings.

While included countries have reliable individual surveillance programmes, national nutrition surveillance is at risk of being unsustainable since many programmes are reliant on external funding. Most national health officials expressed the need for stable funding mechanisms. Past experiences in establishing and expanding nutrition surveillance programmes suggest that collaborating with a wider range of partners with similar interests, priorities and information, under the guidance of a governmental body, fosters a more sustainable nutrition surveillance.^170^

Not unique to south-east Asia, most other countries implement internationally linked multicountry survey platforms, for example, Demographic and Health Surveys, Multiple Indicator Cluster Surveys and the WHO STEPwise approach.^171^ Such large-scale programmes can be valuable for enhancing national governments’ capacity to map national trends and collect standardized, internationally comparable, high-quality nutrition data. However, the intervals of these surveys are 4–5 years, which prevents timely monitoring and evaluation. Furthermore, the surveys generally require external technical and financial support and can be time-intensive to implement.^7^

We found limited monitoring of food environment and broader food system indicators within nutrition surveillance programmes led by national health authorities. Similarly, non-health governmental bodies also inadequately monitor these indicators to directly back the national nutrition agenda. Ideally, countries’ local health and/or nutrition agencies should possess the expertise and capacity to transform broader environment data into comprehensible nutrition-sensitive indicators and metrics. This information should then be used to devise, monitor and enhance nutrition interventions and policies.^172^ Integration of data from nutrition surveillance programmes with other sectoral data can also be valuable as it reduces labour, time and economic costs.^125^ There is substantial data monitoring within south-east Asia on food environments and systems through agricultural and/or industry surveys; commercial databases; academic studies; and routine national surveillance data (food supply and prices).^79^ Such data, in combination with other data sources, are presently transformed by international initiatives and research groups into interpretable nutrition-sensitive indicators such as nutritious food affordability.^79^^,^^168^

By incorporating three different literature search strategies, including an academic database search, grey literature search, and consultations with senior officials of national health authorities where possible, we ensured that we produce a comprehensive review. In addition, including 14 national health and nutrition officials knowledgeable about the surveillance programmes in their respective countries as co-authors further reinforces the credibility of our review findings. Another strength of the study is the use of CDC’s integrative and adaptive framework to obtain a more comprehensive picture of the current state of nutrition surveillance in south-east Asia.

Our review also has limitations. We mainly focused on nationally representative, government-led and -funded programmes. In every country, nutrition-related data originate from diverse sources, varying in form and format, including commercial databases like Euromonitor, as well as research organizations. Future research should explore other sources to get a more comprehensive picture of the countries’ national nutrition information systems.^173^^,^^174^ For example, in some countries, routine surveillance through health information management systems can be a valuable source of nutrition-related data.^175^ Moreover, due to the large number of countries and programmes included, we limited our assessment to a descriptive analysis, using a simplified version of the CDC evaluation framework for public health surveillance. However, we will report findings from the perspectives of countries’ experts on the state of nutrition surveillance in a separate publication. Future research should explore perspectives of local programme personnel and data users, to gain a fuller picture of the implementation and use of nutrition surveillance programmes.

Box 4 presents the implications of our findings, and suggests avenues for research and development to enhance nutritional surveillance. Efforts to improve the time efficiency, scope and stability of national nutrition surveillance should be encouraged and supported, to allow monitoring and evaluation of malnutrition interventions in these countries. By highlighting the features of active, locally led nutrition surveillance programmes in south-east Asia, we aim to equip policy-makers and researchers worldwide with information to enhance nutrition surveillance globally.

Box 4 **Key research and development priorities for nutritional surveillance programmes in study countries**

- Innovative, cost-effective techniques are needed for timely monitoring of nutrition and dietary outcomes that national authorities can use to accurately track population nutrition, and assess the impact of nutritional interventions.
- To improve representativeness in surveillance programmes, innovative sampling methods are needed to include the most vulnerable populations.
- Integration of intersectoral, nutrition-sensitive data and adapting or adopting well- established food system monitoring instruments (such as International Network for Food and Obesity/Noncommunicable Diseases Research, Monitoring and Action Support)^172^ is needed to strengthen governments’ capacity to assess and monitor characteristics of food environments.
